# Redox state and altered pyruvate metabolism contribute to a dose-dependent metformin-induced lactate production of human myotubes

**DOI:** 10.1152/ajpcell.00186.2023

**Published:** 2023-09-11

**Authors:** Jennifer Maurer, Xinjie Zhao, Martin Irmler, Anders Gudiksen, Nanna S. Pilmark, Qi Li, Thomas Goj, Johannes Beckers, Martin Hrabě de Angelis, Andreas L. Birkenfeld, Andreas Peter, Rainer Lehmann, Henriette Pilegaard, Kristian Karstoft, Guowang Xu, Cora Weigert

**Affiliations:** ^1^Department for Diagnostic Laboratory Medicine, Institute for Clinical Chemistry and Pathobiochemistry, University Hospital Tübingen, Tübingen, Germany; ^2^Key Laboratory of Separation Science for Analytical Chemistry, Dalian Institute of Chemical Physics, The Chinese Academy of Sciences, Dalian, China; ^3^Institute of Experimental Genetics, Helmholtz Munich, Neuherberg, Germany; ^4^Section for Cell Biology and Physiology, Department of Biology, University of Copenhagen, Copenhagen, Denmark; ^5^Centre for Physical Activity Research (CFAS), Rigshospitalet, University Hospital of Copenhagen, Copenhagen, Denmark; ^6^German Center for Diabetes Research (DZD), Neuherberg, Germany; ^7^Chair of Experimental Genetics, Technical University of Munich, Freising, Germany; ^8^Institute for Diabetes Research and Metabolic Diseases, Helmholtz Munich, University of Tübingen, Tübingen, Germany; ^9^Department of Internal Medicine IV, University Hospital Tübingen, Tübingen, Germany; ^10^Department of Clinical Pharmacology, Bispebjerg and Fredriksberg Hospital, University Hospital of Copenhagen, Copenhagen, Denmark

**Keywords:** lactate, metformin, pyruvate dehydrogenase complex, respiration, skeletal muscle

## Abstract

Metformin-induced glycolysis and lactate production can lead to acidosis as a life-threatening side effect, but slight increases in blood lactate levels in a physiological range were also reported in metformin-treated patients. However, how metformin increases systemic lactate concentrations is only partly understood. Because human skeletal muscle has a high capacity to produce lactate, the aim was to elucidate the dose-dependent regulation of metformin-induced lactate production and the potential contribution of skeletal muscle to blood lactate levels under metformin treatment. This was examined by using metformin treatment (16–776 μM) of primary human myotubes and by 17 days of metformin treatment in humans. As from 78 µM, metformin induced lactate production and secretion and glucose consumption. Investigating the cellular redox state by mitochondrial respirometry, we found metformin to inhibit the respiratory chain complex I (776 µM, *P* < 0.01) along with decreasing the [NAD^+^]:[NADH] ratio (776 µM, *P* < 0.001). RNA sequencing and phospho-immunoblot data indicate inhibition of pyruvate oxidation mediated through phosphorylation of the pyruvate dehydrogenase (PDH) complex (39 µM, *P* < 0.01). On the other hand, in human skeletal muscle, phosphorylation of PDH was not altered by metformin. Nonetheless, blood lactate levels were increased under metformin treatment (*P* < 0.05). In conclusion, the findings suggest that metformin-induced inhibition of pyruvate oxidation combined with altered cellular redox state shifts the equilibrium of the lactate dehydrogenase (LDH) reaction leading to a dose-dependent lactate production in primary human myotubes.

**NEW & NOTEWORTHY** Metformin shifts the equilibrium of lactate dehydrogenase (LDH) reaction by low dose-induced phosphorylation of pyruvate dehydrogenase (PDH) resulting in inhibition of pyruvate oxidation and high dose-induced increase in NADH, which explains the dose-dependent lactate production of differentiated human skeletal muscle cells.

## Graphical abstract

**Figure:**
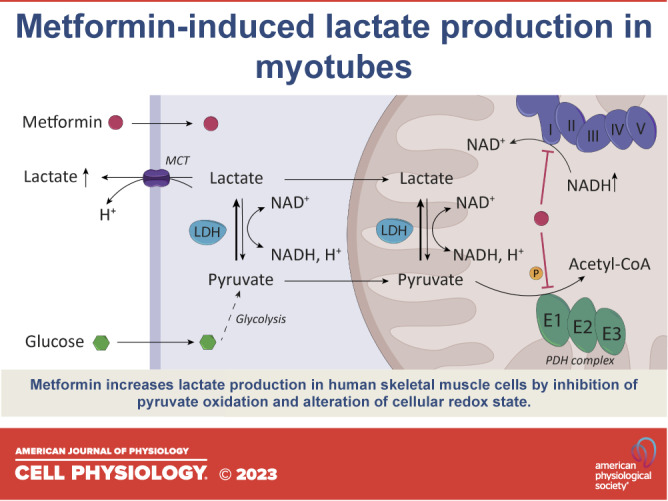

## INTRODUCTION

Since the approval of metformin by the FDA in 1994, the oral antidiabetic drug has become essential in the treatment of type 2 diabetes as first-line therapy and is daily taken by over 150 million people worldwide (1). Patients benefit not only from a blood glucose lowering effect with low risk of hypoglycemia but also from modest weight loss, and cardiovascular and reno-protective effects (2, 3). The reduction of hepatic gluconeogenesis has been recognized as a major blood glucose lowering effect of metformin in patients with type 2 diabetes, and AMP-dependent kinase (AMPK)-dependent and independent pathways have been highlighted as potential contributors to the inhibition of hepatic glucose output (4–6). Moreover, there has been increasing debate as to whether intestinal mechanisms can contribute to the antihyperglycemic and insulin-sensitizing effect of metformin (7, 8). This clearly indicates that the exact mechanism of metformin action is not yet fully understood. Notably, the liver and the gastrointestinal tract are tissues with a high uptake and intraorgan concentration of metformin (9, 10).

Therapeutic doses of metformin are generally well tolerated. But in some cases, patients suffer from acidosis as life-threatening side effect. The risk for metformin-induced acidosis is increased by comorbidities that reduce the renal clearance of metformin or the hepatic uptake of lactate (11). Although acidosis associated with increased glycolysis in metformin users is a rare event (12), metformin therapy has been associated with slightly higher plasma lactate levels in the physiological range (13). Some but not all studies showed a slight increase in plasma lactate levels after initiation of metformin therapy or in comparison to non-metformin users (14). This little-noticed effect of metformin has recently received more attention based on the lactate-induced activation of its receptor G-protein coupled receptor 81 (GPR81), which has been hypothesized to contribute to beneficial effects of the drug on the cardiovascular system, the kidney, and the brain (15). Although this novel concept needs experimental validation, the origin and the mechanisms responsible for the metformin-induced raise in plasma lactate have also not been unraveled. In particular, skeletal muscle has been poorly investigated in this context although it comprises on average 40% of the total body mass (16) and has a high capacity to produce lactate (17). Liver and the gastrointestinal tract are tissues with a high uptake rate and concentration of metformin, but slow accumulation of metformin was also detected in skeletal muscle (9, 10). Therefore, we aim to elucidate the dose-dependent effect of metformin on lactate production using primary human skeletal muscle cells and to identify molecular mechanisms that contribute to the increase in lactate production in human skeletal muscle with metformin treatment. As opposite effects on mitochondrial respiration in other cell systems have been reported for low- and high metformin concentrations (18), it is of particular interest to investigate pharmacological and suprapharmacological doses of metformin to cover the range from therapeutic to toxic concentrations.

## MATERIALS AND METHODS

### Cell Culture

Primary human myoblasts used within this work derive from a pool of muscle biopsies of vastus lateralis collected within a recently published study at the preintervention timepoint of 26 healthy donors with overweight or obesity (15 females, 11 males) (19). All study participants gave written informed consent. The study protocol was in accordance with the declaration of Helsinki and was approved by the ethics committee of the University of Tübingen. The study was registered at Clinicaltrials.gov as trial number NCT03151590. In brief, after collagenase digestion CD56-positive myoblasts were isolated using magnetic cell sorting (MACS) microbeads (Milteny Biotech, Bergisch Gladbach, Germany) as described by Hoffmann et al. (20). Myoblasts were seeded on nongelling thin-layer GelTrex (Thermo Fisher Scientific, Schwerte, Germany) coated surfaces with 10,000 cells/well of a 6-well plate, 100,000 cells/10-cm dish or 150,000 cells/15-cm dish and grown in cloning medium [α-MEM:Ham’s F-12 (1:1), 20% FBS, 1% chicken extract, 2 mM glutamine, 100 U/mL penicillin, 100 μg/mL streptomycin, and 0.5 μg/mL amphotericin B] until 90% confluency was reached. Myotube fusion was induced (*day of differentiation 0*; *D0*) by providing fusion medium [α-MEM, 2 mM glutamine, 100 U/mL penicillin, 100 μg/mL streptomycin, and 0.5 μg/mL amphotericin B, BSA-coupled fatty acids (50 µM palmitate, 50 µM oleate) and 100 µM carnitine; final concentration of 1.61 g/L BSA in fusion medium]. Medium was changed three times a week. Metformin (metformin hydrochloride; Cayman Chemical, Ann Arbor, MI) treatment was performed with fresh medium 48 h before harvest (*D3*) in the concentrations as stated. Harvest and analysis were performed at *D5*.

### Lactate Production and Glucose Consumption

Lactate and glucose were measured in 500 µL centrifuged supernatant collected before harvest at the routine site by enzymatic-based methods (lactate: lactate oxidase; glucose: hexokinase; ADVIA Chemistry XPT, Siemens Healthineers, Erlangen, Germany). As the fusion medium does not contain lactate, measured amount of lactate is considered as cellular lactate production. For calculation of cellular glucose consumption, the detected amount of glucose in the supernatant was subtracted from the glucose concentration (5.5 mM) of the fusion medium. Cellular glucose uptake was determined using the Glucose Uptake-Glo Assay (Promega, Madison, WI) following manufacturers protocol. Metformin was present for 48 h including the 3-h fasting period in glucose-free DMEM media. Incubation with 10 mM 2-deoxyglucose was performed for 1 h at room temperature. Luminescence was measured using the GloMax Multi Detection System (Promega Corporation, Madison, WI) after 1-h incubation at room temperature with the provided detection reagent.

### Quantification of Creatine Kinase and Lactate Dehydrogenase

Creatine kinase (CK) and lactate dehydrogenase (LDH) were quantified in 500 µL centrifuged supernatant collected before harvest by routine methods (LDH: kinetic enzymatic-based method; CK: modified kinetic method of Szasz; ADVIA Chemistry XPT, Siemens Healthineers, Erlangen, Germany).

### LC-MS/Intracellular Concentrations of Metformin

At *D5*, medium from 10-cm dishes was removed and cells were washed twice with 5 mL cold isotonic saline solution on ice. One milliliter (1 mL) of 80% ACN containing 8 ng/mL of metformin-d6 hydrochloride (Sigma-Aldrich, St. Louis, MO) as internal standard was added. Cells were lysed in the TissueLyser II (Qiagen, Hilden, Germany) for 2 min at 20 Hz, followed by 15 min centrifugation at 14,000 *g*, 4°C. The supernatant was divided into two aliquots of 450 µL and vacuum dried overnight (20 mbar, 1,500 rpm, RVC 2-33 IR, Martin Christ, Osterode am Harz, Germany) together with the remaining cell pellet. The cell pellet was resuspended in 1 mL of protein solubilization buffer (5 M urea, 2 mM thiourea, 15 mM DTT, 2% CHAPS in H_2_O) and the total amount of protein was quantified by Bradford assay (Bio-Rad Laboratories, Hercules, CA). Intracellular concentrations of metformin were normalized to amount of total protein. The dried supernatant was dissolved in 50 µL of 10% ACN/H_2_O. For the quantification of metformin, 0.1 µL was injected in a Shimadzu LCMS-8050 mass spectrometer. The separation was performed on a 5 cm × 2.1 mm Discovery HS F5 3 μm column. The mobile phase consisted of 0.1% formic acid in water (A) and 0.1% formic acid in ACN (B). The gradient elution was changed from 10% eluent B to 98% eluent B within the first 2 min, then kept for 2 min, and finally returned back to 10% eluent B for another 2 min. Flow rate was set to 0.3 mL/min and the column temperature was kept at 50°C. The limit of detection was 1.4 ng/mL metformin.

### High-Resolution Respirometry/Seahorse

Myoblasts were cultured directly on the Seahorse assay plates (Seahorse XFe24 FluxPak, Agilent, Santa Clara, CA) with 20,000 cells/well as described earlier. At *D5*, cells were washed according to manufacturer’s protocol with the indicated Seahorse assay medium (DMEM, 10 mM glucose, 2 mM l-glutamine, 1 mM pyruvate or 1 mM lactate). After calibration of the Seahorse XFe24 Analyzer (Agilent Technologies, Santa Clara, CA), the assay was performed using freshly prepared substrates diluted in Seahorse assay medium: 10 µM oligomycin (Port 1), 20 µM FCCP (Port 2), and 5 µM rotenone and 5 µM antimycin A (Port 3). After the assay, cells were lysed in 50 µL of RIPA buffer and protein content was determined in 10 µL of the lysate by bicinchoninic acid (BCA) assay (Pierce Biotechnology, Waltham, MA) in duplicates for normalization of the oxygen consumption rate (OCR). The Seahorse measurements were performed on 3–5 replicates for each condition using myoblasts from six individual donors. The calculation of the respiration at different respiratory states and visualization of bioenergetic phenotypes was carried out according to the manufacturer’s protocol.

### High-Resolution Respirometry/Oroboros

At *D5*, medium was removed from 15-cm dishes and cells were washed twice with 10 mL of DPBS on ice. One milliliter (1 mL) of cold Mir05 buffer (0.5 mM EGTA, 3 mM MgCl_2_, 60 mM lactobionic acid, 20 mM taurine, 10 mM KH_2_PO_4_, 20 mM HEPES, 110 mM d-sucrose, 1 g/L essentially fatty acid free BSA, pH 7.1) was added. Cells were carefully scraped and cell suspension from each dish was divided into equal parts for duplicate measurement. A volume of 100 µL of each duplicate was separated for determination of protein content in 10 µL by BCA assay (Pierce Biotechnology, Waltham, MA) to normalize the determined O_2_ flux. Remaining 400 µL of the samples were pelleted (13,000 *g*, 5 min, 4°C) and supernatant was removed. The pellet was resuspended in 100 µL of Mir05 and injected into the measurement chamber of an Oxygraph-2k (Oroboros Instruments, Innsbruck, Austria), which was pre-equilibrated with 2 mL of Mir05 at 37°C. After reaching a plateau, 12 µM digitonin was used for cell permeabilization. In the following, stepwise injection of the substrates with a final concentration of 1.3 mM malate, 2.5 mM ADP, 500 µM octanoylcarnitine, 5 mM pyruvate, 10 mM glutamate, 10 mM succinate, 10 µM cytochrome C, FCCP titration (1 µM steps until maximum), 1 μM rotenone, 5 μM antimycin A was performed always waiting for a plateau in between. Reoxygenation was performed before O_2_ < 120 µM, which occurred usually during FCCP titration.

### NAD^+^/NADH Assay

The intracellular concentrations of NAD^+^ and NADH were determined separately using the NAD/NADH-Glo Assay (Promega, Madison, WI) following manufacturers protocol. In brief, myotubes on 6-well plates at *D5* were washed with 1 mL of cold DPBS. Cells were suspended by scraping in 100 µL of DPBS and 10 µL of each sample were transferred into a white 96-well plate for the following acidic (NAD^+^) or basic (NADH) treatment to separately determine the two analytes. After 30 min incubation at room temperature, luminescence was measured using the GloMax Multi Detection System (Promega Corporation, Madison, WI). Relative light units (RLUs) were normalized to protein content based on BCA Assay (Pierce Biotechnology, Waltham, MA) of 10 µL cell suspension.

### Immunoblotting of Cell Lysates

Protein lysates were prepared in cold RIPA buffer (25 mM Tris pH 7.6, 150 mM NaCl, 0.1% SDS, 0.5% NaDOC, 1% Triton X100) containing cOmplete EDTA-free protease inhibitor (Roche Diagnostics, Rotkreuz, Switzerland) and phosphatase inhibitor (1 mM NaF, 0.5 mM sodium pyrophosphate, 1 mM β-glycerophosphate, 1 mM sodium orthovanadate). Protein concentration was determined by BCA assay (Pierce Biotechnology, Waltham, MA) and samples were heated in Laemmli sample buffer (60 mM Tris-HCl, 12.5% glycerol, 1% SDS, 5% β-mercaptoethanol, 0.1% bromophenol blue) for 5 min to 95°C or 50°C for mitochondrial protein detection. Sodium dodecyl sulfate polyacrylamide (7.5–15%) gradient gel electrophoresis and semidry electroblotting [transfer buffer: 48 mM Tris, 39 mM glycine, 0.0375% sodium dodecyl sulfate, and 20% (vol/vol) methanol] were performed using PVDF membranes (Immobilon FL, Merck Millipore, Burlington, MA). NET buffer (150 mM NaCl, 50 mM Tris/HCl, pH 7.4, 5 mM EDTA, 0.05% Triton X-100, and 0.25% gelatin) was used for blocking. Proteins were detected using the antibodies listed in Table 1 (primary, overnight at 4°C; secondary, 2 h at room temperature) on an Odyssey scanner (LI-COR Biosciences, Lincoln, NE).

**Table 1.** Primary and secondary antibodies used for immunoblotting of cell lysates

Target	Catalogue Number	Supplier	Dilution in Net-G	Host/Antibody Type
*Primary antibodies*				
NDUFB8	ab110242	abcam	1:2,000	Mouse monoclonal
Citrat Synthase	ab96600	abcam	1:1,000	Rabbit polyclonal
GAPDH	ab8245	abcam	1:20,000	Mouse monoclonal
PDH-E1α	sc-377092	Santa Cruz	1:500	Mouse monoclonal
PDH phospho (pSer232)	AP1063	Calbiochem	1:500	Rabbit polyclonal
PDH phospho (pSer293)	ab92696	abcam	1:400	Rabbit polyclonal
PDH phospho (pSer300)	AP1064	Merck	1:500	Rabbit polyclonal
*Secondary antibodies*				
IRDye Donkey@Rabbit 800CW	925-32213	LI-COR	1:10,000	Donkey anti-rabbit IgG
IRDye Goat@Mouse 680RD	925-68070	LI-COR	1:20,000	Goat anti-mouse IgG

### RNA Isolation and RNA Sequencing

Isolation of total RNA was performed according to the manufactures protocol using the RNeasy Mini Kit (Qiagen, Hilden, Germany) after cells were washed with 1 mL of cold DPBS. The RNA concentration was determined by NanoDrop2000 (Thermo Fisher Scientific, Schwerte, Germany). The RIN number was determined using the Agilent RNA 6000 Nano Kit (Agilent Technologies, Santa Clara, CA) as additional quality control. A total amount of 300 ng RNA was provided to BGI Tech Solutions (Hongkong) for commercial sequencing analysis using the DNBSEQ Eukaryotic Strand-specific Transcriptome Resequencing protocol, resulting in cleaned, adapter-free paired-end data. Reads were aligned to the GRCh38.103 genome [STAR v2.7.9a, (21)], and gene-level read counts were obtained (summarizeOverlaps, mode = “Union,” package GenomicAlignments v.1.30.0). Ensembl gene IDs were annotated based on the Ensembl database using Biomart (genome version GRCh38.p13). RNA-seq data have been submitted to the GEO database at NCBI (GSE229658).

### Metformin Effects in Human Muscle Biopsies

Human muscle biopsies were collected as part of a crossover study described by Pilmark et al. (22). In brief, 15 healthy young men (age 23.7 ± 0.6; BMI 22.3 ± 2.0, V̇o_2peak_ 3.5 ± 0.6 L/min) underwent two treatment periods with either placebo or metformin (gradually increased over 8 days to 2,000 mg/day) each lasting 17 days separated by a 4-day washout period in between. Protein lysates were prepared from skeletal muscle biopsies, collected from m. quadriceps femoris, vastus lateralis, before and immediately after an acute bout of exercise (45-min cycling, 70% V̇o_2peak_) at *day 17* of each treatment period. Detailed description of the immunoblotting protocol can be found in Refs. 22 and 23. Primary antibodies against phosphoPDH Ser232 (Calbiochem, AP1063), and phosphoPDH Ser293, phosphoPDH Ser300, and PDH-E1α protein (all provided by Prof. Graham Hardie, Dundee, Scotland) and species-specific horseradish peroxidase-conjugated immunoglobulin secondary antibodies (Dako, Glostrup, Denmark) were used. Simultaneous to the biopsy collection, blood samples were drawn and used for determination of blood lactate levels (ABL 7 series; Radiometer, Denmark).

### Data Analysis and Statistics

Initial data were collected using the software for the corresponding devices as provided by the manufactures: Microplate Manager (v.6.3, Bio-Rad Laboratories, Hercules, CA), Wave (v.2.6.3, Agilent Technologies, Santa Clara, CA), DatLab7 (v.7.4.0.4, Oroboros Instruments, Innsbruck, Austria), Image Studio Lite (v.5.2.5, LI-COR Biosciences, Lincoln, NE), NanoDrop2000 (v.1.5, Thermo Fisher Scientific, Schwerte, Germany), 2100expert (v.B.02.11.SI811, Agilent Technologies, Santa Clara, CA). Data were collected in Excel (Microsoft, Redmond, WA) that was used for normalization and other calculations. If indicated, raw data of myotubes of each donor were normalized. Statistical analyses were performed using R [v.4.2.1, (24)]/RStudio (v.2022.02.3). A gene-level differential expression analysis using “DESeq2” [v.1.32.0, (25)] was carried out on the RNA sequencing data after removing low expressed genes across all samples (>60 sum of total counts). The *P* values attained by the Wald test were corrected for multiple testing using the Benjamini and Hochberg method (adj.*P*). Significant genes were defined by adj.*P* < 0.1. Gene sets were filtered for average counts >5 in at least one group per comparison. The functional enrichment analysis was performed using g:Profiler (26) using g:SCS multiple testing correction method applying significance threshold of 0.05. *T* tests were used to compare metformin-treated groups against control and Spearman rank correlation was used for correlation analysis. ANOVA followed by Tukey honestly significant difference (HSD) tests was performed for multiple pairwise-comparison analysis. A *P* value <0.05 was considered statistically significant. Further statistical parameters and significance levels are shown in the figure legends. Graphs were plotted using the R packages “ggplot2” (v.3.3.5), “ggsignif” (v.0.6.3), and “ggrepel” (v.0.9.1). Data are presented as means ± standard deviation (SD). The indicated *n* number represents the number of biopsies derived from individual donors included in the experiment.

## RESULTS

### Cellular Uptake of Metformin

**Figure 1.**
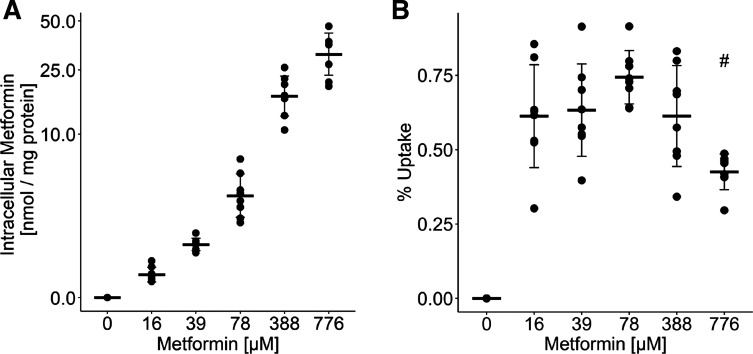
Cellular uptake of metformin in human myotubes. *A*: intracellular concentrations of metformin were determined with LC-MS in cell lysates of primary human myotubes after 48 h of treatment with indicated concentrations of metformin and were normalized to the total protein content of the lysate. *B*: percentage of supplemented metformin that was taken up by the myotubes. Individual data points are displayed (*n* = 8 donors); bars represent means ± SD; *t* test between groups: #different from all other metformin treated groups *P* < 0.05.

First of all, we quantified the amount of metformin in cell lysates of metformin-treated myotubes by LC-MS to study cellular uptake. Metformin was detected in all samples except the control group (Fig. 1*A*). Treatment with metformin in a low micromolar range corresponding to pharmacological blood concentrations found in metformin-treated patients resulted in an intracellular concentration of 0.66 ± 0.21 nmol/mg protein and 1.65 ± 0.22 nmol/mg protein after 48 h of treatment with 16 and 39 µM metformin, respectively, whereas the highest metformin concentration resulted in 32.38 ± 8.46 nmol/mg protein. In percentage of the total supplemented metformin dose, an uptake from 0.43 ± 0.06% to 0.74 ± 0.08% was detected in the cell lysates (Fig. 1*B*). Treatment with 776 µM metformin resulted in a significantly lower percentual uptake (*P* < 0.05), which suggests intracellular saturation. The data are in a similar range with concentrations reported in primary rat hepatocytes with 1.3, 2.3, and ≥10 nmol/mg protein after treatment with 100, 200, and 1,000 µM metformin (27) and 1–2 nmol/mg protein after treatment with 100 and 200 µM metformin in mouse hepatocytes (28), suggesting that primary myotubes have a similar uptake rate as primary hepatocytes.

### Metformin Increases Lactate Production in Myotubes

**Figure 2.**
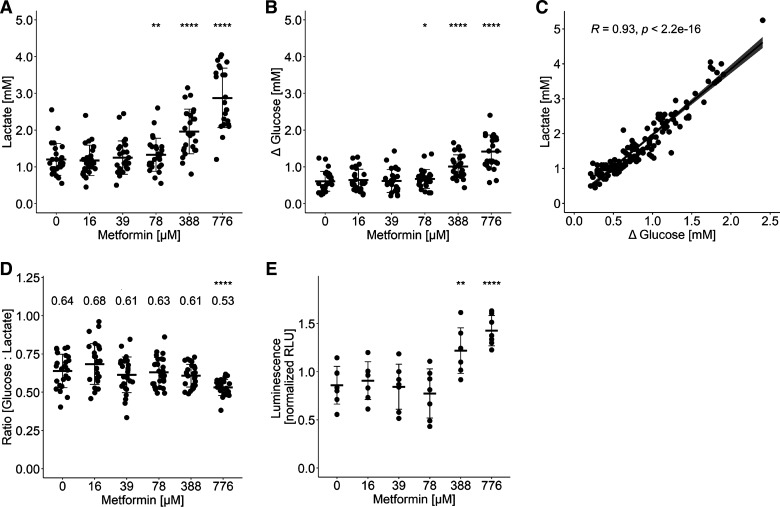
Metformin enhances lactate production and glucose consumption in human myotubes. The concentrations of lactate and glucose were determined in the supernatant collected immediately before harvesting the myotubes after 48 h of treatment with metformin. The measured amount of lactate is counted as cellular lactate production after 48 h (*A*). Glucose consumption (*B*) was calculated as the difference between the concentration in the medium and the supernatant. All data points represent the mean value of technical duplicates (*n* = 26 donors); bars represent means ± SD; paired *t* test against 0 µM metformin: ns *P* > 0.05, *P < 0.05, **P < 0.01, ****P < 0.0001. *C*: Spearman correlation of lactate production with cellular glucose consumption. *D*: ratio between glucose consumption and lactate production (*n* = 26 donors); bars represent means ± SD; paired *t* test against 0 µM metformin: ns *P* > 0.05, *P < 0.05, **P < 0.01, ****P < 0.0001. *E*: cellular uptake of glucose was determined based on a bioluminescent assay using 2-deoxyglucose (2-DG). All data points represent the mean value of technical duplicates after donor normalization (*n* = 7 donors); bars represent means ± SD; *t* test against 0 µM metformin: **P < 0.01, ****P < 0.0001. RLU, relative light units.

Next, we investigated which concentrations of metformin lead to an increase in lactate production of primary human myotubes over 48 h of treatment. Metformin enhanced lactate production and secretion in a dose-dependent manner (≥78 µM, *P* < 0.01, Fig. 2*A*). Meanwhile, glucose consumption increased (≥78 µM, *P* < 0.05, Fig. 2*B*) and was positively correlated (Fig. 2*C*) with the increase in lactate production. The stochiometric ratio of glucose:lactate ranges between 0.68 and 0.53 (Fig. 2*D*) and shows a significant decrease for the treatment using 776 µM metformin (*P* < 0.0001). These data underline glycolysis as driver of the increased lactate production forcing lactate secretion into the supernatant. In addition, the treatment with metformin also increased glucose uptake assessed as uptake of 2-deoxyglucose over 60 min (≥388 µM, *P* < 0.01, Fig. 2*E*). To rule out cell damage causing cellular lactate leakage by the metformin treatment, we quantified the concentrations of creatine kinase (CK) and lactate dehydrogenase (LDH) in the supernatant. For both parameters, we found no significant changes compared with the untreated control group (Supplemental Fig. S1).

### Metformin Alters Cellular Redox State by Inhibition of Complex I Respiration

**Figure 3.**
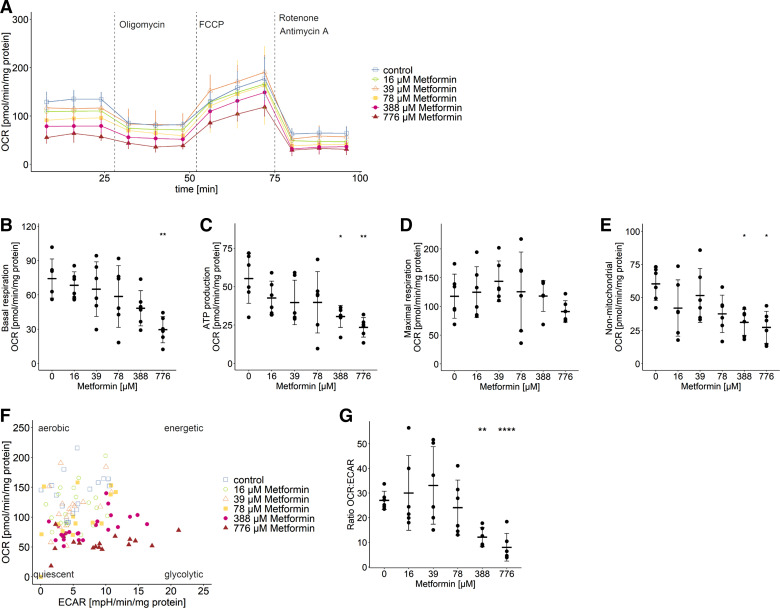
Metformin blunts mitochondrial respiration and induces a shift toward glycolytic phenotype. *A*: the dose-effect relationship on mitochondrial respiration was determined using the seahorse apparatus. Respiratory states were quantified as follows: basal respiration as oxygen consumption rate (OCR) at the beginning of the protocol, when glucose, pyruvate, and glutamine are present (*B*), ATP production after addition of oligomycin (*C*), maximal respiration after addition of FCCP (*D*), and non-mitochondrial oxygen consumption after addition of rotenone and antimycin A (*E*). All data points represent the mean value of technical replicates of 3–4 (*n* = 6 donors); bars represent means ± SD; ANOVA and Tukey HSD test were performed for multiple pairwise-comparison between the means of groups: ns *P* > 0.05, *P < 0.05, **P < 0.01. *F*: energy map visualizing basal normalized OCR and extracellular acidification rate (ECAR) reflecting four metabolic phenotypes: aerobic (predominantly mitochondrial respiration), glycolytic (predominantly glycolysis), energetic (both pathways), and quiescent (low activity via either metabolic pathway). All data points represent the mean value of technical replicates of 3–4 (*n* = 6 donors). *G*: ratio of basal normalized OCR to ECAR in dependence of indicated metformin dose. All data points represent the mean value of technical replicates of 3–4 (*n* = 6 donors); bars represent means ± SD; paired *t* test against 0 µM metformin: ns *P* > 0.05, **P < 0.01, ****P < 0.0001.

Searching for molecular mechanisms responsible for the metformin-induced increase in lactate production, we focused on pathways increasing either intracellular pyruvate or NADH, thereby shifting the equilibrium of the lactate dehydrogenase (LDH) reaction and forcing lactate production. Therefore, we investigated the influence of metformin on the mitochondrial respiration of myotubes. Using the seahorse system, we covered the full range of metformin concentrations used in the previous experiments (Fig. 3, *A–E*). After treatment with high metformin concentrations for 48 h, basal respiration (776 µM, *P* < 0.01, Fig. 3*B*), ATP production (≥388 µM, *P* < 0.05, Fig. 3*C*), and non-mitochondrial respiration (≥ 338 µM, *P* < 0.05, Fig. 3*E*) were reduced whereas the maximal respiration was unaffected (Fig. 3*D*). As the equilibrium of LDH reaction is sensitive to changes in lactate, pyruvate, and NADH/NAD^+^, we wanted to rule out that the observed metformin-induced changes in mitochondrial respiration are influenced by the pyruvate concentration of 1 mM in assay medium. Therefore, we compared the influence of either pyruvate or lactate as provided substrates in addition to glucose and glutamine on mitochondrial respiration (Supplemental Fig. S2). We found no differences in the respiratory states between the different composed assay media. On top, metformin reduced basal respiration and ATP production in the presence of 1 mM lactate or pyruvate similarly (Supplemental Fig. S2, *B* and *C*). The analysis of the basal normalized oxygen consumption rate (OCR) versus extracellular acidification rate (ECAR) allows a relative bioenergetic phenotyping. With higher doses of metformin, myotubes shift from an aerobic to a more glycolytic phenotype (Fig. 3*F*), which is also represented by the decrease in OCR:ECAR ratio (≥388 µM, *P* < 0.01, Fig. 3*G*).

**Figure 4.**
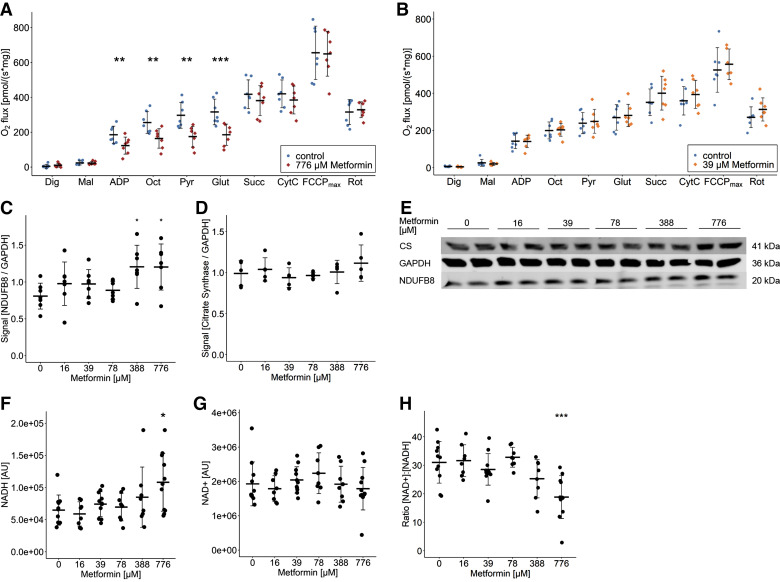
Metformin alters cellular redox state by inhibition of complex I respiration. Mitochondrial respiration of permeabilized myotubes was analyzed after treatment with 776 µM (*A*) and 38 µM (*B*) metformin using the Oroboros system. Complex I respiration was determined after addition of malate, ADP, pyruvate, and glutamate. Complex II respiration was initiated by addition of succinate. Maximal uncoupled respiration was determined by titration of FCCP. O_2_ flux was normalized to mg protein lysate. All data points represent the mean value of technical duplicates (*n* = 7 donors); bars represent means ± SD; paired *t* test between both groups: ns *P* > 0.05, **P < 0.01, ***P < 0.001. *C* and *D*: quantification of signal intensity of immunoblots detecting NADH:ubiquinone oxidoreductase subunit B8 (NDUFB8) and citrate synthase (CS) related to glyceraldehyde-3-phosphate dehydrogenase (GAPDH). All data points represent the mean value of technical duplicates after donor normalization (*n* = 7/5 donors); bars represent means ± SD; *t* test against 0 μM metformin: ns *P* > 0.05, *P < 0.05. *E*: representative immunoblots of technical duplicates of each condition of one donor. The total cellular amount of NADH (*F*) and NAD^+^ (*G*) was determined simultaneously using a luciferase assay and the ratio was calculated (*H*). All individual data points are displayed (*n* = 11 donors); bars represent means ± SD; paired *t* test against 0 μM metformin: *P < 0.05, ***P < 0.001.

To gain a deeper understanding of which respiratory complexes were targeted by metformin, we measured mitochondrial respiration during the consecutive injection of substrates in the oroboros system. The application of ADP and octanoylcarnitine, but also further addition of pyruvate and of glutamate showed significantly lower O_2_ flux in myotubes treated with 776 µM metformin (*P* < 0.01, Fig. 4*A*). All these substrates fuel complex I respiration. The increase in O_2_ flux after addition of ADP is due to the activity of mitochondrial malic enzyme 2 (ME2) that produces pyruvate (20). As soon as succinate, which is a sole complex II substrate, was added, the difference in the O_2_ flux of the control group and the metformin-treated group disappeared and thus, maximal oxidative phosphorylation and electron transfer system capacity (after addition of FCCP) were not affected by metformin. In line with the results of the seahorse analysis, the inhibition in complex I respiration was not detected when myotubes were treated with the lower dose of 39 µM metformin (Fig. 4*B*). Notably, complex I subunit NDUFB8 used as marker for complex I abundance was slightly increased after metformin treatment (≥ 388 µM, *P* < 0.05, Fig. 4*C*), whereas citrate synthase (CS) (Fig. 4*D*) as a marker protein of mitochondrial content was unaffected. This suggests that the reduced complex I respiration is not due to changes in mitochondrial mass or reduced complex I abundance. We also analyzed the effect of metformin on the differentiation of myotubes, which influences the abundance of mitochondrial enzymes (20). We did not detect any differences in fast- and slow-twitch myosin heavy chain fiber-type used as differentiation markers at the protein level (Supplemental Fig. S3, *A–C*). The inhibition of complex I was paralleled by an increase in NADH and subsequently, a decrease in the ratio of [NAD^+^]:[NADH] (776 µM, *P* < 0.001, Fig. 4, *F–H*). This suggests, that high metformin concentrations inhibit complex I respiration and alter the cellular redox state, which contributes to the metformin-induced lactate production. However, the data do not explain the lactate formation observed with lower metformin concentrations of 78 or 388 µM.

### Metformin Induces the Inhibition of Pyruvate Oxidation through Phosphorylation of PDH

**Figure 5.**
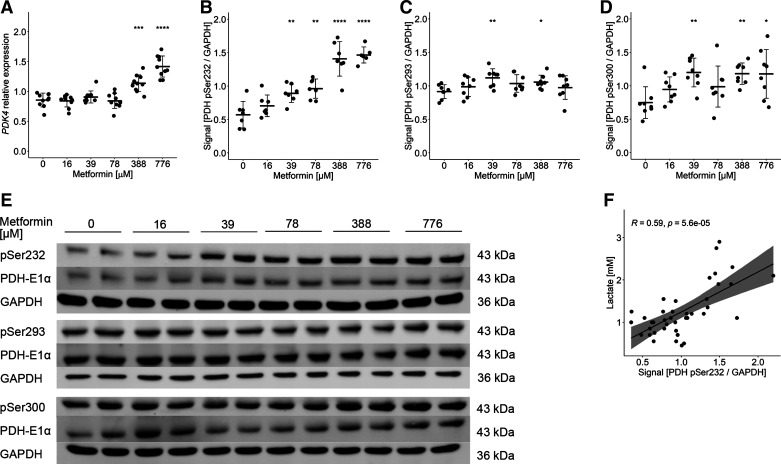
Metformin induces the inhibition of pyruvate oxidation through phosphorylation of pyruvate dehydrogenase (PDH). *A*: relative expression of *PDK4* by RNA sequencing. All individual data points are displayed (*n* = 10 donors); bars represent means ± SD; adjusted *P* values (Benjamini–Hochberg): ***P < 0.001, ****P < 0.0001. Quantification of signal intensity of immunoblots detecting phosphorylation at Ser232 (*B*), Ser293 (*C*), and Ser300 (*D*) of the PDH complex subunit E1α (PDH-E1α). All data points represent the mean value of technical duplicates after donor normalization (*n* = 8 donors); bars represent means ± SD; *t* test against 0 μM metformin: *P < 0.05, **P < 0.01, ***P < 0.001, ****P < 0.0001. *E*: representative immunoblots of technical duplicates of each condition of one donor. *F*: Spearman correlation of the phosphorylation of the PDH complex on Ser232 site with the increase in lactate production.

In a next step, we focused on mechanisms that could lead to accumulation of pyruvate. RNA sequencing data of myotubes treated for 48 h with 16–776 µM showed no enrichment of regulated genes in glucose or pyruvate metabolism (Supplemental Fig. S4). Searching the results for metformin-regulated enzymes and proteins involved in pyruvate conversion according to GO terms pyruvate transport (GO:0006848), pyruvate dehydrogenase activity (GO:0004738), pyruvate dehydrogenase complex (GO:0045254), and Mitocarta3.0 pathway pyruvate metabolism, the data revealed an upregulation of *PDK4* mRNA under metformin treatment for 48 h (≥388 µM, *P* < 0.001, Supplemental Table S1 and Fig. 5*A*). Other pyruvate dehydrogenase kinase (PDK) isoforms, subunits of the pyruvate dehydrogenase complex, or mitochondrial pyruvate carriers showed no comparable changes in mRNA levels (Supplemental Table S1). PDKs are responsible for phosphorylation of pyruvate dehydrogenase (PDH), thereby inhibiting the enzyme and preventing conversion of pyruvate to acetyl-CoA. Using immunoblots, we demonstrated that phospho-site Ser232 of PDH responded in a dose-dependent manner to metformin treatment of human myotubes and showed an increased phosphorylation state (≥39 µM, *P* < 0.01, Fig. 5*B*). Phosphorylation at site Ser293 and Ser300 were also increased after treatment with 39 µM, which in contrast to phosphorylation of Ser232 was not further increased with higher doses of metformin (≥39 µM, *P* < 0.01, Fig. 5, *C* and *D*). Phosphorylation of one of these three sites is sufficient to inhibit the activity of the PDH complex (29). Notably, the phosphorylation of Ser232 of PDH showed a significant positive correlation with lactate production (Fig. 5*F*). These data provide a basis for the conclusion that the effects of PDH phosphorylation, and hence PDH activity, on the LDH equilibrium may be the mechanism behind metformin-mediated regulation of lactate production specifically in the lower micromolar range of metformin.

### Metformin and Exercise Increase Blood Lactate Levels In Vivo

**Figure 6.**
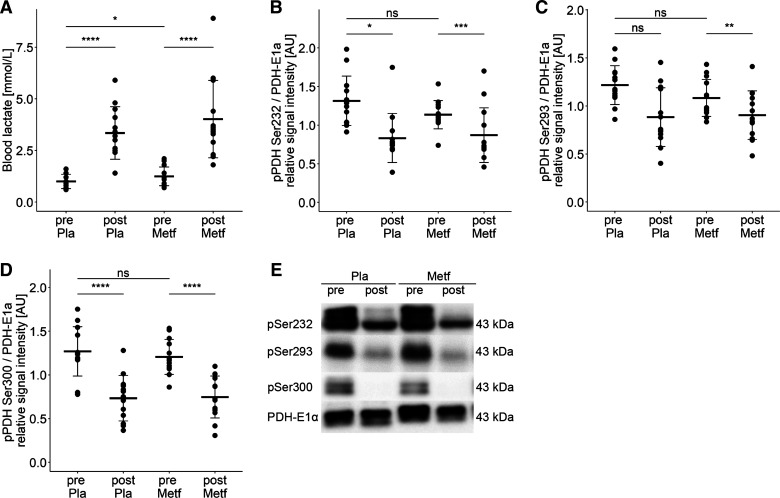
Metformin and exercise increase blood lactate levels in vivo. *A*: fasting blood lactate levels were determined at the same timepoint as biopsies were taken. All individual data points are displayed (*n* = 14 donors), bars represent means ± SD; paired *t* test between groups: *P < 0.05, **P < 0.01, ***P < 0.001, ****P < 0.0001. Quantification of signal intensity of immunoblots detecting phosphorylation at Ser232 (*B*), Ser293 (*C*), and Ser300 (*D*) of the pyruvate dehydrogenase (PDH) complex subunit E1α (PDH-E1α) from human muscle biopsies taken immediately before (pre) and after (post) an acute bout of exercise. Pla, Placebo; Metf, Metformin. All individual data points after donor normalization are displayed (*n* = 13 donors); bars represent means ± SD; *t* test between groups: ns *P* > 0.05, *P < 0.05, **P < 0.01, ***P < 0.001, ****P < 0.0001. *E*: representative immunoblot showing samples of one donor.

Finally, we asked whether the metformin effects on pyruvate metabolism found in primary human myotubes can be also observed in human skeletal muscle. We investigated muscle biopsies that were collected in a recently published study (22), where 15 young healthy men received metformin and placebo treatment each for 17 days in a crossover study design. At the end of each treatment period the subjects underwent an acute bout of exercise (45 min, 70% V̇o_2max_). Muscle biopsies as well as venous blood samples were taken immediately before and after the exercise intervention. Blood lactate concentrations were slightly higher in the metformin arm before exercise (1.00 ± 0.34 vs. 1.25 ± 0.43, *P* < 0.05, Fig. 6*A*). After finishing the exercise bout, blood lactate increased in both treatment groups with no significant impact of metformin. Analyzing the phosphorylation of the PDH complex on the three serine sites, we found no significant differences between placebo and metformin treatment (Fig. 6, *B–E*) but a strong reduction in phosphorylation of all three serine sites immediately after the acute bout of exercise (*P* < 0.05). With these results, we provide evidence that metformin treatment with pharmacological doses elevates blood lactate levels in healthy humans, but the in vivo data do not support metformin-mediated inhibition of the PDH complex as a mechanism for an increased lactate production in human skeletal muscle during metformin treatment.

## DISCUSSION

Here, we report that treatment with metformin increases lactate secretion of primary human myotubes in a dose-dependent manner, showing the effect at concentrations of 78 µM and above. The positively correlated increase in glucose consumption suggests that the increased secretion is due to increased glycolysis and lactate production, which is underlined by a shift in the OCR to ECAR ratio. Consistent with this, we found significantly higher glucose uptake rates in myotubes treated with higher doses of metformin. Other studies showing a metformin-induced increase in lactate production in myotubes (30, 31) used concentrations of 2–10 mM metformin. Markedly lower concentrations of 90 µM metformin were sufficient to increase lactate production in isolated rat soleus muscle (32), which is in a similar range compared with our data. Based on a review by Huang et al. (14), it must be stated that plasma lactate levels of patients without comorbidities are at most slightly elevated. However, a community-based study (13) in patients with type 2 diabetes showed elevated plasma lactate levels under metformin treatment by a mean of 0.28 mmol/L. Similarly, we show a slight significant increase of blood lactate using a maximum of 2,000 mg metformin/day in young, healthy volunteers. However, considering the regularly reported systemic metformin concentrations in humans treated with pharmacological doses of metformin in a range of 5–40 µM (5), and the concentrations needed to stimulate lactate production in isolated muscle and cultured myotubes, a contribution of lactate production in skeletal muscle to systemic lactate concentrations in metformin-treated patients appears unlikely. Consistent with this, 16 wk of metformin treatment of patients with type 2 diabetes did not increase lactate release from skeletal muscle (33). When reaching slightly higher systemic metformin concentrations, as it can be foreseen in patients with reduced renal function or compromised hepatic metabolism (11, 34), a contribution of skeletal muscle to the increase in blood lactate is possible.

Based on these considerations two questions arise: what are the underlying mechanisms of metformin-induced lactate production and which other tissues can be responsible for the increase in systemic lactate concentrations in metformin-treated patients.

**Figure 7.**
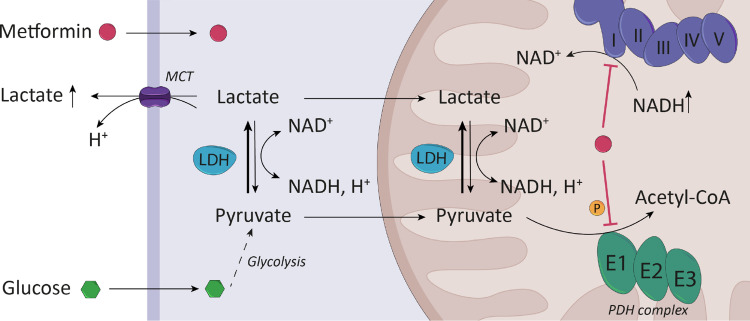
Metformin-induced lactate production in human myotubes. Metformin increases lactate secretion in human myotubes by shifting the equilibrium of the lactate dehydrogenase (LDH) reaction and thus forcing lactate production. One driver of this effect is the increase in glycolysis. In addition, metformin alters the cellular redox state through the inhibition of respiratory chain complex I. Increased phosphorylation of the pyruvate dehydrogenase (PDH) complex inhibits pyruvate oxidation, which also contributes to the observed increase in lactate secretion.

As potential mechanism for a shift in the LDH reaction toward lactate production in myotubes, we provide evidence for increased intracellular pyruvate due to inhibition of the PDH reaction as well as increased NADH by reduced complex I respiration (Fig. 7). We show that metformin did not reduce mitochondrial protein abundance, which is consistent with published data (35, 36) and suggests that reduced complex I respiration is not due to changes in mitochondrial content or reduction in complex I proteins. Although the inhibitory effect of metformin on complex I respiration has been reported previously in studies using different species, tissues, and cell lines, the ongoing debate is more about the underlying mechanisms and the concentrations used (37, 38). Most of the in vitro studies reporting inhibitory effects of metformin on complex I respiration observe this effect using concentrations in the millimolar range (32, 39–42) whereas an opposite effect was observed for low micromolar concentrations in hepatocytes (18), but not in C2C12 myoblasts (41). Our data support the previous results showing that suprapharmacological metformin concentrations are necessary for inhibition of complex I with no effect of pharmacological concentrations in the low micromolar range. In isolated mitochondria and isolated complex I preparations, even higher concentrations than in intact cells had to be used to observe an inhibitory effect (38, 39, 43). In one of the few human studies, ex vivo metformin titration of permeabilized muscle fibers from healthy subjects confirms the dose-dependent inhibitory effect on complex I respiration (42), using albeit concentrations above 3 mM. The exact mechanism behind complex I inhibition has not yet been elucidated but binding of metformin and other biguanides to the amphipathic region of the complex was reported (44). The unsolved question is whether intramitochondrial metformin concentrations in cell culture or in vivo are sufficient to block complex I activity by direct binding. Earlier work has suggested that metformin accumulates within mitochondria (40). However, mitochondrial accumulation of metformin was not detected in hepatocytes, where most of the intracellular metformin was found in the cytoplasmic fraction. In that study, inhibition of complex I was observed with 500 µM metformin similar to our data (18).

Inhibition of complex I respiration results in a decrease in cellular NAD^+^/NADH ratio and following, in increased lactate production. This effect has been recently demonstrated in hepatocytes and C2C12 cells using rotenone, a widely used complex I inhibitor (45). Our results of inhibited complex I respiration are supported by the finding that metformin increased cellular NADH levels and consequently, cellular NAD^+^/NADH ratio was lowered. We attribute the fact that we see no effect on NAD^+^ level to the circumstance that the ratio of NAD^+^ to NADH is high [a factor of 20–30 in our human myotubes, 10–20 were reported in skeletal muscle (46)] and detection of a small decline in NAD^+^ may be limited by assay sensitivity. To conclude, if metformin concentrations are sufficient to inhibit complex I by either mitochondrial accumulation and direct interaction with complex I or by yet unknown mechanisms, this forces lactate production by the decreased cellular NAD^+^/NADH ratio. However, complex I inhibition was not observed in the low micromolar range of metformin, whereas 78 µM metformin induced lactate production and higher glucose consumption in the myotubes. Thus, other mechanisms must be of relevance, to explain the lactate-inducing effects of lower metformin concentrations.

One such mechanism is inhibition of pyruvate conversion to acetyl-CoA by phosphorylation of PDH complex. We found increased PDH-E1α phosphorylation at the three serine sites Ser232, Ser293, and Ser300 using low metformin concentrations (≥39 µM) and the degree of phosphorylation at Ser232 correlates positively with the amount of lactate produced. An impact of metformin on pyruvate metabolism is supported by RNA sequencing data of human skeletal muscle biopsies obtained from older subjects with impaired glucose tolerance [based on oral glucose tolerance test (OGTT)] after 6 wk of metformin treatment within a placebo-controlled crossover study (47). Pathway analysis of differentially expressed genes revealed an association with pyruvate metabolism and regulation of PDH complex. Increased transcript levels of *PDK4* as a prevalent PDK isoform in skeletal muscle and myotubes (48–50) provide an explanation for increased PDH phosphorylation using 388 and 776 µM metformin. Notably, inhibition of pyruvate oxidation and the increased NADH/NAD^+^ ratio would not only have consequences for the cytosolic equilibrium of the LDH reaction, but also for the mitochondrial conversion of lactate to pyruvate and thus reducing lactate oxidation (Fig. 7) (51).

In human skeletal muscle biopsies, when analyzing the phosphorylation of the PDH complex on the three serine sites, we found no metformin-induced effect, but a strong reduction in phosphorylation of all three serine sites immediately after the acute bout of exercise. This is consistent with previous reports showing an immediate increase in PDH activity after exercise (for review, see Ref. 52), which is associated with reduced PDH phosphorylation in human skeletal muscle (53). PDH activation is initiated due to increased sarcoplasmic Ca^2+^ that activates PDH dephosphatases and is supported by the inhibition of PDKs by pyruvate, ADP, and NADH (52). It is not surprising that the metformin treatment did not influence the exercise-regulated PDH phosphorylation status in human skeletal muscle that is ensured by multiple pathways. Similarly, it is not unexpected that the difference in blood lactate levels in both treatment groups was neutralized after acute exercise, because exercise is a potent stimulus for muscle lactate release. However, we could also not observe a metformin effect on PDH phosphorylation in the rested state, which again suggests that the metformin concentrations reached in vivo in healthy subjects using pharmacological doses of metformin do not enhance lactate production in skeletal muscle via the proposed mechanism of PDH complex inhibition.

The relevance of experimental concentrations of metformin used in in vitro experiments for translating the results to the in vivo situation has always been a point of discussion, but comparable intracellular in vivo data are rare. In human myotubes, we found intracellular concentration ranging from 0.66 ± 0.21 to 32.38 ± 8.46 nmol/mg protein depending on the supplied amount of metformin. These data are in a similar range with concentrations reported in primary rat hepatocytes (27, 28), suggesting that primary myotubes have a similar uptake rate as primary hepatocytes. This appears to be in contrast to in vivo data of metformin uptake in humans. Whole body PET dosimetry of ^11^C-metformin demonstrated slow accumulation with discrete tracer uptake in skeletal muscle, whereas it was much more pronounced in liver and kidneys (9). The intestinal tract also shows considerable uptake of metformin (10), and metformin has been shown to stimulate intestinal glycolysis and lactate production resulting in an increase of lactate in the portal vein (54). Thus, slight increases in blood lactate levels under metformin treatment can be due to intestinal lactate release in the circulation. To provide a basis for a more accurate interpretation of results obtained in skeletal muscle cells, the specific determination of intramyofibrillar metformin concentrations in humans would be an important piece of information. In summary, we identified dose-dependent mechanisms by which metformin can increase lactate production in primary human myotubes. Given the high concentrations of metformin required to achieve complex I inhibition, this can only be of relevance in a situation of a toxic tissue accumulation of the drug. The in vitro metformin-mediated inhibition of pyruvate oxidation by phosphorylation of PDH requires considerably lower metformin concentrations. Although the in vivo importance of this metformin-induced regulation of pyruvate metabolism in skeletal muscle needs further experimental validation, the mechanism could also play a role in tissues with high uptake of metformin such as the intestine and contribute to blood lactate levels in metformin-treated patients.

## DATA AVAILABILITY

RNA-seq data have been submitted to the GEO database at NCBI (GSE229658).

## SUPPLEMENTAL DATA

10.6084/m9.figshare.22762121 Supplemental Figs. S1–S4 and Table S1: https://doi.org/10.6084/m9.figshare.22762121.

## GRANTS

The work was partially supported by the German Federal Ministry of Education and Research (BMBF) to the German Center for Diabetes Research under Grant No. 01GI0925, by the Mobility Programme of the Sino-German Center for Research Promotion under Grant No. M-0257, the Key Foundation 21934006 from the National Natural Science Foundation of China. The in vivo study was supported by a grant from Aase and Ejnar Danielsen Foundation, a grant from the Research Foundation of Rigshospitalet (Denmark), and by King Christian X Foundation.

## DISCLOSURES

No conflicts of interest, financial or otherwise, are declared by the authors.

## AUTHOR CONTRIBUTIONS

J.M. and C.W. conceived and designed research; J.M., X.Z., A.G., and Q.L. performed experiments; J.M., X.Z., M.I., A.G., Q.L., and T.G. analyzed data; J.M., A.G., H.P., and C.W. interpreted results of experiments; J.M. prepared figures; J.M. and C.W. drafted manuscript; J.M., M.I., A.G., N.S.P., H.P., K.K., G.X., and C.W. edited and revised manuscript; J.M., X.Z., M.I., A.G., N.S.P., Q.L., T.G., J.B., M.H.d.A., A.B., A.P., R.L., H.P., K.K., G.X., and C.W. approved final version of manuscript.
